# Multimorbidity of Allergic Conditions in Urban Citizens of Southern China: A Real-World Cross-Sectional Study

**DOI:** 10.3390/jcm12062226

**Published:** 2023-03-13

**Authors:** Ya-Ting Li, Ming-Hui Hou, Ya-Xin Lu, Pei-Ran Chen, Zhen-Yuan Dai, Li-Fen Yang, Ping-Ping Zhang, Guo-Wei Xiong, Zi-Feng Liu, Qi-Lin Zhou, Jing Su, Yun Cheng, Yu-Qi Zhou, Jin Tao, Xue-Kun Huang, Min Dai, Kun Zhang, Min Zhou, Qin-Tai Yang, Pei-Ying Feng, Zhuang-Gui Chen

**Affiliations:** 1 Department of Allergy, The Third Affiliated Hospital of Sun Yat-sen University, Guangzhou 510630, China; 2 Department of Pediatrics, The Third Affiliated Hospital of Sun Yat-sen University, Guangzhou 510630, China; 3 Department of Clinical Data Center, The Third Affiliated Hospital of Sun Yat-sen University, Guangzhou 510630, China; 4 Department of Dermatology, The Third Affiliated Hospital of Sun Yat-sen University, Guangzhou 510630, China; 5 Department of Pulmonary and Critical Care Medicine, The Third Affiliated Hospital of Sun Yat-sen University, Guangzhou 510630, China; 6 Department of Gastroenterology, The Third Affiliated Hospital of Sun Yat-sen University, Guangzhou 510630, China; 7 Department of Otolaryngology-Head and Neck Surgery, The Third Affiliated Hospital of Sun Yat-sen University, Guangzhou 510630, China; 8 Department of Traditional Chinese Medicine, The Third Affiliated Hospital of Sun Yat-sen University, Guangzhou 510630, China

**Keywords:** allergic disease, multimorbidities, Southern China, allergy management

## Abstract

Background: Extensive knowledge of allergic multimorbidities is required to improve the management of allergic diseases with the industrialization of China. However, the demography and allergen distribution patterns of allergic multimorbidities in China remain unclear, despite the increasing prevalence of allergies. Methods: This was a real-world, cross-sectional study of 1273 outpatients diagnosed with one or more allergic diseases in Guangzhou, the most populated city of southern China, with leading industrial and commercial centers, between April 2021 and March 2022. Seven allergic diseases (allergic rhinitis (AR), asthma (AS)/cough variant asthma (CVA), atopic dermatitis (AD)/eczema, food allergy (FA), allergic conjunctivitis (AC), drug allergy (DA), and anaphylaxis) were assessed. Positive rates of sensitization to different allergens were measured using an allergen detection system of the UniCAP (Pharmacia Diagnostics, Sweden) instrument platform to compare the groups of allergic multimorbidities against a single entity. Results: There were 659 (51.8%) males and 614 (48.2%) females aged from 4 months to 74 years included in the analysis. The study participants who were diagnosed with allergic diseases had an average of 1.6 diagnoses. Overall, 46.5% (592 of 1273) of the patients had more than one allergic condition, and allergic rhinitis was the most common type of multimorbidity. Women were more likely to suffer from an allergic disease alone, whereas allergic multimorbidities were more likely to be diagnosed in men (*p* = 0.005). In addition, allergic multimorbidities were common in all age groups, with an incidence ranging from 37.1% to 57.4%, in which children and adolescents were more frequently diagnosed with allergic multimorbidities than adults (18–60 years old) (all *p* < 0.05). Allergic multimorbidity was observed throughout the year. A difference in the positive rate of allergens sensitization and total immunoglobulin E (tIgE) levels between different allergic multimorbidities was observed. Conclusions: Allergic multimorbidities were very commonly found in nearly half of all patients with allergies. The proportion of allergic multimorbidities varied with the type of disease, sex, age, and allergen distribution pattern. These findings may help clinicians to develop “One health” strategies for the clinical management of allergic diseases.

## 1. Introduction

Over the past few decades, with the acceleration of industrialization, increase in environmental pollution, changes in lifestyle and dietary structure, and continuous increase in allergen exposure, allergy diseases affect 30–40% of the global population and cause a major disease burden worldwide [1,2,3,4], with an estimated 300 million patients with bronchial asthma (AS), 400 million patients with allergic rhinitis (AR), 200 million to 250 million patients with food allergy (FA), 150 million patients with drug allergy (DA), and numerous patients with allergic conjunctivitis (AC), angioneurotic edema, urticaria, eczema, eosinophilic diseases, insect allergy, and anaphylactic shock [5]. Concomitantly, allergy is listed by the World Health Organization (WHO) as one of the 6 major chronic diseases in the 21st century.

Although some birth cohort studies have been initiated to better understand this high incidence, the studies have almost exclusively targeted a single allergic entity [6,7]. A few prospective cohort studies have examined the multimorbidity of allergic diseases, but mostly in patients in early childhood [8,9,10,11]. Pols et al. found that AR, allergic asthma, and AD have a 9.8 times higher risk of occurring simultaneously in children [12]. A cross-sectional study of combined European, birth, cohort data by the EU-funded MeDALL Consortium showed that the coexistence of asthma, rhinitis, and eczema in the same child at ages 4 and 8 years was more prevalent than expected by chance alone [13]. However, the progression of these coexisting conditions into childhood and adulthood in patients with allergic diseases has not been investigated in China.

In addition, many allergic conditions, which influence and aggravate each other, can occur in the same patient [14,15]. Presently, in China, most patients still need to visit different clinical specialties, such as otorhinolaryngologists, respiratory specialists, dermatologists, gastroenterologists, or pediatric specialists, for different allergic symptoms, which brings great disadvantages to the management of allergic diseases and increasing medical expenses. We chose Guangzhou, the capital city of China’s most populated province, with a population of 18 million, as well as the largest city in southern China with a high incidence of allergic diseases. The city has established a few allergy centers/departments, which provide one-stop comprehensive diagnosis and treatment services for patients with allergic diseases. The purpose of this study was to investigate the epidemiology of multimorbidity of various allergic diseases in allergic patients in order to update allergologists with the knowledge to develop “One health” strategies to treat allergic multimorbidities [16].

## 2. Materials and Methods

### 2.1. Study Design

This was a real-world, cross-sectional study involving patients with allergic diseases.

### 2.2. Ethics Statement

This study was conducted in compliance with the protocol approved by the Ethics Committee of the Third Affiliated Hospital of Sun Yat-sen University. Written informed consent was obtained from the patients (for patients aged ≥18 years) and their guardians (for patients aged <18 years) before enrollment. This information was obtained after explaining the nature and possible consequences of the study.

### 2.3. Participants

The study participants were outpatients at an Allergy Department of the Third Affiliated Hospital of Sun Yat-sen University, between April 2021 and March 2022. Only participants who fulfilled the following criteria were included: (1) a local resident of Guangzhou (a permanent resident of Guangzhou or living in Guangzhou for more than 6 months) and (2) diagnosed with at least one or more allergic diseases by clinical specialists. To avoid the impact of some diseases on the level of IgE, patients with a diagnosis of parasitic infection, deep fungal infection, immunodeficiency disease, or malignant tumors were excluded. Patients with ambiguous or suspicious diagnoses were excluded from the study. In total, 1273 patients were diagnosed with one or more allergic diseases, and all patients agreed to participate in this study.

### 2.4. Clinical Data Acquisition 

We defined seven different allergic diseases according to the international consensus of diagnostics or expert consensus (detailed in Table S1), including AR [17], AS/cough variant asthma (CVA) [18], AD/eczema [19,20], FA [21], AC [22], drug allergy (DA) [23], and anaphylaxis [24]. Patients were diagnosed and treated by two independent clinical specialists from the Department of Allergy, who then screened them for the above seven allergic diseases according to the inclusion and exclusion criteria and completed the electronic medical record files. Information on age, sex, and family history, as well as blood samples, were collected for further analysis.

### 2.5. Allergen-Specific Immunoglobulin E Antibody or Total Immunoglobulin E Detection.

Specialists selected the specific immunoglobulin E (sIgE) antibody of the inhaled and/or ingested allergens and/or serum total immunoglobulin E (tIgE) to be tested according to clinical requirements. A 3–4 mL sample of fresh, peripheral, venous blood was collected from each patient, clotted at room temperature for 30 min, and centrifuged at 4000 rpm for 5 min. Serum was separated and stored at 4 °C until further examination. The UniCAP (Pharmacia Diagnostics, Uppsala, Sweden) instrument platform, which provides an automated test system incorporating sample and reagent handling, washing, reading, calculation, integrated allergen coupled processing, and printing, was used to detect serum sIgE or tIgE. All tests were performed in a controlled ImmunoCAP environment maintained at a constant temperature. Data are stored, and integrated daily calibration is carried out automatically and compared with the stored values, providing the UniCAP system with high standards of precision, reproducibility, and fast processing [25]. The serum sIgE level was expressed as a concentration of kUA/L. The results of sIgE were graded from Class 0 to 6 according to the concentration gradient provided by UniCAP system: Class 0, lower than 0.35 kUA/L; Class 1, 0.35–0.69 kUA/L; Class 2, 0.70–3.49 kUA/L; Class 3, 3.5–17.49 kUA/L; Class 4, 17.5–49.9 kUA/L; Class 5, 50.0–100.0 kUA/L; Class 6, higher than 100.0 kUA/L. An sIgE measure of 0.70 kUA/L or more was defined as a positive detection.

### 2.6. Statistical Analysis 

Continuous data are described using the median (interquartile range [IQR]). The Wilcoxon rank-sum test was used for the comparison between two groups of independent samples, the Kruskal–Wallis test was used for the comparison between multiple groups, and the Benjamini Hochberg corrected Dunn’s test was used for multiple comparisons. The categorical data were described as counts (percentages), and the chi-square test was used to compare the distribution differences between groups. Statistical analysis and visualization were performed by R-3.6.2 (R Foundation for Statistical Computing, Vienna, Austria). All results were statistically significant at *p* < 0.05.

## 3. Results

### 3.1. Characteristics of Patients with Allergic Diseases

**Figure 1:**
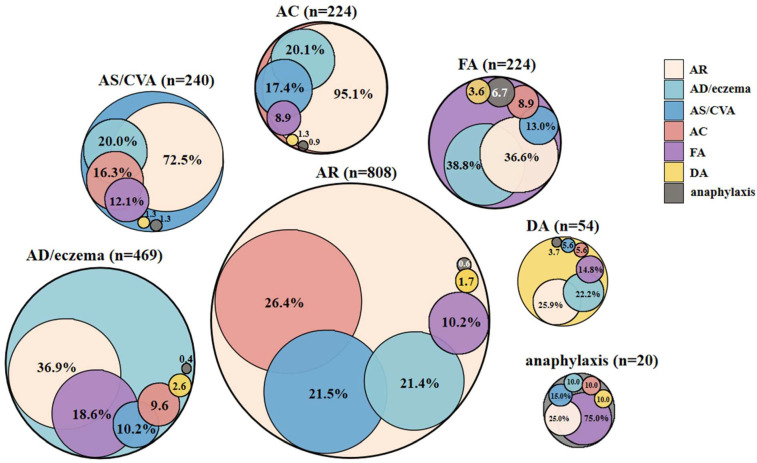
The pattern of allergic conditions in the 1273 patients. Physician-diagnosed allergic rhinitis (AR), asthma (AS)/cough variant asthma (CVA), atopic dermatitis (AD)/eczema, food allergy (FA), allergic conjunctivitis (AC), drug allergy (DA), anaphylaxis, and the overlap of the diagnoses in the study. For example, 26.4% of patients with physician-diagnosed AR also have AC diagnosis, 21.5% AS/CVA, 21.4% AD/eczema, 10.2% FA, 1.7% DA, and 0.6% anaphylaxis.

A total of 1273 patients with allergic diseases were included in the analysis. There were 659 (51.8%) males and 614 (48.2%) females aged 4 months to 74 years, with a median age of 13.0 years [IQR, 6.0–28.0 years]. The allergic diseases identified, in descending order of prevalence, were as follows: AR (*n* = 808, 63.5%), AD/eczema (*n* = 469, 36.8%), AS/CVA (*n* = 240, 18.9%), FA (*n* = 224, 17.6%), AC (*n* = 224, 17.6%), DA (*n* = 54, 4.2%), and anaphylaxis (*n* = 20, 1.6%). The study participants diagnosed with allergic diseases had an average of 1.6 diagnoses (range 1–6) (shown in Table 1). More than one allergic condition was observed in 46.5% (*n* = 592) of patients, and 11.1% (141 of 1273) of patients had three or more diagnoses of allergic diseases. Anaphylaxis had the highest incidences of multimorbidity (*n* = 20, 100.0%), 75.0% of which coexisted with food allergies, and DA had the lowest incidences of multimorbidity (51.9%, 28 of 54) (allergic drugs details shown in Table S2). AR was the most common multimorbidity, followed by 95.1% of the patients with AC, 72.5% with AS/CVA, and 25.0–36.9% of those with other conditions reporting AR. Anaphylaxis was the least common multimorbidity; however, it was present in 0.4–6.7% of patients with other allergic conditions (shown in Figure 1). A total of 47 phenotypes of allergic conditions were observed in this study (shown in Figure S1). The top three allergic multimorbidities, in descending order of prevalence, were AR+AC (*n* = 139), AR+AS (*n* = 94), and AR+AD (*n* = 86), in total accounting for 53.9% (319 of 592) of all the allergic multimorbidities.

### 3.2. Impact of Sex on Patients with Allergic Diseases

The incidence rates of allergic multimorbidities in males and females were 50.4% (332 of 659) and 42.3% (260 of 614), respectively. In general, females were more likely to have a single allergic disease, whereas allergic multimorbidities were more frequently diagnosed in males (*p* = 0.005), which was also observed in AS/CVA (*p* = 0.001) and AD/eczema (*p* = 0.004). However, demographic variables, such as sex patterns, differed among the allergic multimorbidity groups, for example, more males had AR than females. However, no significant gender difference was found between AR and their multimorbidities (*p* = 0.841). (Shown in Table 2).

### 3.3. Impact of Age on Patients with Allergic Diseases

**Figure 2:**
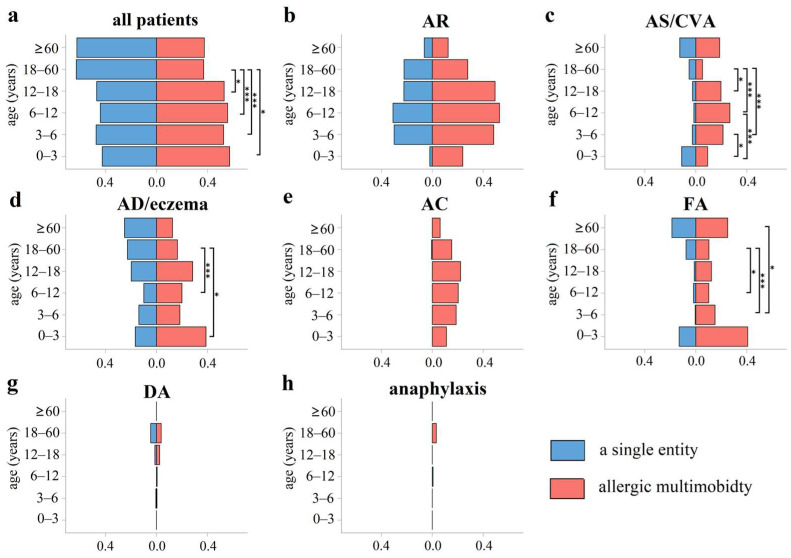
The proportion of allergic multimorbidities and a single entity by age. (**a**) All patients; (**b**) AR; (**c**) AS/CVA; (**d**) AD/eczema; (**e**) AC; (**f**) FA; (**g**) DA; (**h**) anaphylaxis. In general, allergic multimorbidities were more common in children’s groups and adolescents than the adult group (18–60 years old). Children of preschool age (3–6 years), school age (6–12 years), and the adolescent group (12–18 years) were more susceptible to AS/CVA multimorbidities than the adult group (18–60 years). The incidence of AD/eczema alone in the adult group (18–60 years old) was higher than that in school-age (6–12 years old) children and infants (0–3 years old) (both *p* < 0.05). In addition, the incidence of FA multimorbidities was lower in the adult group (18–60 years old) than that in school age (6–12 years old) and preschool age (3–6 years) children (both *p* < 0.05). *: *p* < 0.05; ***: *p* < 0.001.

The patients were divided into 6 groups according to age: (1) infant group (0–3 years), 54 cases; (2) preschool age group (3–6 years), 224 cases; (3) school age group (6–12 years), 324 cases; (4) adolescent group (12–18 years), 81 cases; (5) adult group (18–60 years), 574 cases; and (6) older adult group (≥60 years), 16 cases. In general, allergic multimorbidities were common in all age groups, with incidences ranging from 37.1 to 57.4% in allergic patients. In addition, adults had an average of 1.4 diagnoses (range 1–5), less than all groups of children and adolescents, with an average of 1.7 to 1.8 diagnoses (range 1–6) (all *p* < 0.05). All children and adolescent groups were more frequently diagnosed with allergic multimorbidities than the adult group (18–60 years old) (all *p* < 0.05) (shown in Figure 2). Similar results were also found in different allergic diseases, such as children of preschool age (3–6 years), school age (6–12 years), and the adolescent group (12–18 years) were more susceptible to AS/CVA multimorbidities than the adult group (18–60 years) (all *p* < 0.05), and the incidence of AD/eczema multimorbidities in the adult group (18–60 years old) was lower than that in school-age (6–12 years old) children and infants (0–3 years old) (both *p* < 0.05) (shown in Figure 2).

### 3.4. Seasonality

The number of people diagnosed with allergic diseases each month was stable, but slightly lower from April to June, the beginning of summer in Guangzhou, China. However, no significant differences in the frequency of allergic multimorbidities were observed throughout the year, as well as between any single entity and their multimorbidities (shown in Figure S2).

### 3.5. Spectrum of Allergen Sensitization and tIgE in Patients with Allergic Diseases

During the investigation, 95.9% (1221 of 1273) of patients with allergic diseases were detected for tIgE, and 77.4% (985 of 1273) of patients were detected for allergen sIgE. Samples from the same patients and those from patients who received or had received allergen immunotherapy and/or used biological agents were excluded.

In general, the positivity rate of inhaled allergens was 82.2% (794 of 966), and the top 5 allergens, in descending order of prevalence, were as follows: *D. farinae* (77.7%, 738 of 950), *D. pteronyssinus* (76.8%, 730 of 950), cockroaches (20.2%, 132 of 654), dander (15.3%, 86 of 562), and pollen (5.0%,21 of 418). The positivity rate of ingested allergens was only 38.3% (181 of 473), with the allergens identified as follows (the top 5, in descending order of prevalence): shrimp (22.0%, 75 of 341), milk (21.0%, 85 of 404), crab (18.0%, 61 of 338), egg white (15.6%, 61 of 391), and peanuts (9.5%, 7 of 74).

The spectrum of allergen sensitization in patients with allergic diseases and their comorbidities is summarized in Table 3, Table 4 and Table S3. Patients with AR, AS/CVA, or AD/eczema multimorbidities had a significantly higher positivity rate for *D.* pteronyssinus or *D.* farinae than those with a single allergic disease (AR, 86.9% vs. 80.5% and 88.0% vs. 81.3%; AS/CVA, 91.5% vs. 47.1% and 91.4% vs. 44.1%; AD/eczema, 83.0% vs. 49.0% and 83.9% vs. 49.5%; *D. pteronyssinus* and *D. farinae*, all *p* < 0.05). Similar results were obtained between AS/CVA or AD/eczema and their multimorbidities for cockroach allergens (shown in Table 3). The positive rate of ingested allergens, such as egg white, milk, wheat, shrimp, or crab, in AD/eczema multimorbidities was found to be higher than AD/eczema alone (all *p* < 0.05) (shown in Table 4).

**Figure 3:**
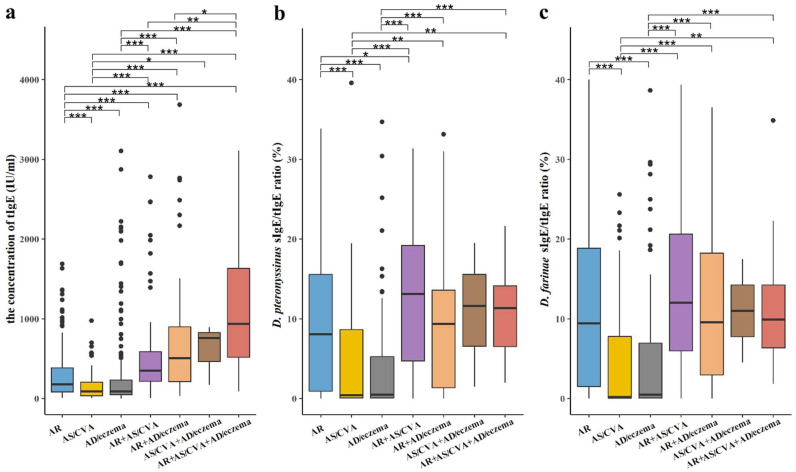
Serum concentration of tIgE and mite sIgE/tIgE ratio in patients with AR, AS, AD, and their multimorbidities. (**a**) Serum concentration of tIgE in patients with AR+AS/CVA+AD/eczema was higher than AR+AS/CVA; Serum concentrations of tIgE of AR or AS/CVA were lower than their multimorbidities; Serum concentration of tIgE of AD/eczema was lower than AR and AR multimorbidities, and AR higher than AS/CVA. (**b**) *D. pteronyssinus* sIgE/tIgE ratio was higher in patients with upper airway allergic multimorbidities, such as AR co-occurring with AS/CVA or AD/eczema, and lower in patients only have AS/CVA or AD/eczema. The ratio in AR was higher than in AS/CVA or AD. (**c**) *D. farinae* sIgE/tIgE ratio pattern was similar to *D. pteronyssinus*. *: *p* < 0.05; **: *p* < 0.01; ***: *p* < 0.001.

The median concentration of tIgE was 216 IU/mL [IQR, 86.4–519], and in 70.7% of the patients (863 of 1221), it exceeded 100 IU/mL. Among the latter, the tIgE level of 18 patients exceeded 5000 IU/mL, and these patients were all diagnosed as AD/eczema, with or without its multimorbidities. When allergic conditions were refined into AR+AS/CVA+AD/eczema, AR+AS/CVA, AR+AD/eczema, AS/CVA+AD/eczema, AR, AD/eczema, and AS/CVA (complications excluded), the median concentration of tIgE between groups was statistically significant (shown in Figure 3a).

We further explored the relationship between mite sIgE of *D. pteronyssinus* or *D. farinae* and tIgE (sIgE/tIgE ratio) in the above seven allergic conditions. The results showed that the *D. pteronyssinus* sIgE/tIgE ratio was higher in patients with upper airway multimorbidities, such as AR co-occurring with AS/CVA or AD/eczema, and the AR entity was higher than AS/CVA or AD/eczema (all *p* < 0.05). The *D. farinae* sIgE/tIgE ratio was similar to that in *D. pteronyssinus* (shown in Figure 3b,c).

## 4. Discussion

In this study, we demonstrated allergic multimorbidities with the industrialization of China to provide a current reference for the comprehensive management of allergic diseases. We chose the outpatients at the Allergy Department, Third Affiliated Hospital of Sun Yat-sen University, to carry out this research, which is a large platform jointly built by multiple disciplines, gathering specialists from otorhinolaryngology, respiratory, dermatology, gastroenterology, pediatrics, traditional Chinese Medicine, and other related clinical departments. It is a famous allergy center in Southern China that provides “one-stop” accurate diagnosis and high-quality, comprehensive treatment for a large number of mildly allergic patients and invites well-known experts in the field to join in the “cloud consultation” mode for severely allergic patients to formulate the best treatment plan. In contrast to the previous study of the Finnish pharmacy survey [14], the diagnosis of allergic conditions of patients was confirmed by specialists in this study, not “patient-reported”, which has a higher accuracy. In addition, the study lasted for a whole year to avoid the selection bias of seasonality.

Among the 1273 patients with allergies, approximately half (46.5%, 592 of 1273) were positive for more than 1 allergic disease, and even 11.1% (141 of 1273) of the patients had 3 or more diagnoses of allergic conditions. Although all types of allergic diseases can be regarded as single, multimorbidity is a common phenomenon. A cross-sectional study of allergic diseases in 1154 college students aged 18–26 years showed that the comorbidity rate of 2 or more (≥2) allergic diseases was 7.9% [26]. Research has suggested that chronic sinusitis with nasal polyps (CRSwNP) [7] and FA also have a higher risk of multimorbidity with asthma [8]. This suggests that allergic multimorbidities are present in a consistent proportion of patients with allergic diseases, which warrants national awareness of their diagnosis and management.

According to our results, the prevalence of allergic multimorbidities was higher in males than in females, whereas single allergic diseases were more frequently detected in females. Some studies have shown that the prevalence of allergic multimorbidities is similar in both sexes in adulthood, whereas single allergic diseases are slightly more common in females than in males. The prevalence rate of girls with AD combined with AR and AD combined with AA was higher than that of boys. In comparison, that of boys with AR combined with AA was higher [15]. Although the influence of sex on allergic multimorbidities needs to be explored further, it has been suggested that the difference may be related to hormone secretion [27]. However, Hohmann C et al. suggested that the incidence of asthma, rhinitis, and respiratory diseases was lower in girls than that in boys. After puberty, the sex difference in the incidence gradually disappeared [28]. A possible explanation for these observations is that the distribution of the spectrum of allergic diseases varies with region, race, and age.

Over the past few decades, “atopic march” was used to describe the natural pathogenesis of these allergic diseases. Longitudinal studies have shown that 1/3 of patients with AD develop asthma, and 2/3 of patients with AD may suffer from AR [29]. Moreover, the clinical guidelines also suggested that atopic eczema in children is a risk factor for developing asthma. However, research on a Ugandan birth cohort found that atopic sensitization increased with age, and atopy or allergic-related diseases (ARD) in early life were associated with later ARD, but the classic “atopic march” was not observed [30]. Hill et al. proposed a more extensive and alternative allergic march model: the occurrence of atopic diseases starts from AD and then develops into any other type of allergic disease; that is, patients may show the development process of allergic diseases without AS or AR. However, according to this model, only 10.5% of children showed progress in the atopic march [31]. It was still controversial that there was no clear evidence to prove the existence of an atopic march, which may be because it is inappropriate to be specific to a certain individual at the population level. In our study, the proportion of patients with allergic multimorbidities varied significantly with age. All children and adolescents were more frequently diagnosed with allergic multimorbidities than adults (18–60 years old). The proportion of patients with two or more allergic conditions did not increase substantially, and the number of diagnoses decreased with age, which reflected that the prevalence of atopic disease did not increase as expected. Our data support the allergic multimorbidity nature of these conditions, but we also lack longitudinal data that would allow us to suggest a driving mechanism, such as the atopic march.

It is known that the incidence rate of some allergic diseases differs based on seasonality, such as seasonal AR or AC. However, our research found that the proportion of patients diagnosed with allergic multimorbidities was stable throughout the year. This may have been related to the fact that mite is the main allergen and mainly leads to perennial AR in Guangzhou.

To better understand the relationship between tIgE and different allergic multimorbidities, we extracted patient data on seven allergic diseases (AR+AS/CVA+AD/eczema, AR+AS/CVA, AR+AD/eczema, AS/CVA+AD/eczema, AR alone, AD/eczema alone, and AS/CVA alone) for analysis. The results showed that the concentration of tIgE in patients with airway allergic multimorbidities was higher than their entities, indicating that tIgE is involved in the pathogenesis of airway allergic multimorbidity. In addition, when airway allergic diseases were complicated with AD/eczema, the tIgE level was higher than that in only airway allergic diseases. Therefore, a previous study indicated that tIgE developed as a Th2 biomarker involved in regulating the Th2 inflammatory response [32]. Dust mites are the main allergens in Southern China [33,34], both in airway and skin allergic diseases. Allergen immunotherapy (AIT) is the only treatment for allergic diseases [35]. The EAACI proposed that the increase in serum sIgE and the symptoms after exposure to allergens are the only criteria for diagnosing allergy at present and are also the selection criteria for AIT [36]. However, the efficacy of AIT based on this standard is only 52–86.9% [37,38,39]. Therefore, many studies have been conducted on these biomarkers. Di Lorenzo et al. suggested that the baseline sIgE/tIgE ratio is the best predictor of the curative effect of AIT [40]. Whether subcutaneous or sublingual desensitization preparations are used, when the sIgE/tIgE ratio is greater than 16.2%, the sensitivity and specificity to dust mites are 97.9% and 93.1%, respectively [41]. However, our results showed that there are obvious differences in this ratio among the different allergic multimorbidities. It is higher when there are co-occurring airway multimorbidities, such as AR co-occurring with AS/CVA, or when combined with allergic skin diseases (AD/eczema), and lower when patients only have AS/CVA or AD/eczema. Therefore, it is necessary to further explore this ratio in clinical applications, particularly in the desensitization treatment of AS/CVA and skin allergic diseases.

Our study had some limitations. First, this study was conducted at a single center, which might have led to an underestimation of the overall diagnostic rate for selected cases. Second, due to the characteristics of real-world research, allergen or tIgE detection for patients was carried out according to the actual clinical condition. Therefore, no unified allergens sIgE or tIgE tests were performed for all enrolled patients. Third, we did not include urticaria in this study, as the etiology of urticaria was complex, part of which was non-allergic, especially for chronic urticaria. However, all of the etiology of urticaria cannot be determined in the actual work in this study. Fourth, there is no specific detection that can be used as the diagnostic criteria for AS in children under 6 years old at present; clinicians mainly evaluate the possibility of children developing persistent AS according to the frequency and severity of symptoms/attacks and whether there are risk factors for AS, so as to determine whether long-term controlled treatment is needed, and to further support or exclude the diagnosis of AS according to the treatment response. In addition, some allergic diseases have different subtypes, such as AD and AS, and more studies are warranted to elucidate the relationship between different subtypes of allergic multimorbidities according to age, sex, and other factors in order to identify clues for potential pathogenesis research.

In summary, to our knowledge, this is the largest real-world, cross-sectional study of allergic multimorbidity in China. As a chronic disease involving multiple systems and fields, the comprehensive management of allergic disease actually coincides with the “One health” strategy, which emphasizes cross-sectoral and multidisciplinary cooperation to promote human health. The management strategies, from the “one airway one diseases” hypothesis for allergic comorbidities, indicate the need for effective management of allergic diseases. It is important that allergologists and healthcare professionals work out strategies for the optimal management of patients with allergic multimorbidities.

## Figures and Tables

**Table 1:** Characteristics of patients with different allergic conditions.

Characteristics	All Patients (n = 1273)	Any Single Allergic Disease (n = 681)	Allergic Multimorbidities (n = 592)	*p* Value
Gender, n (%)				0.005
Male	659 (51.8)	327 (48.0)	332 (56.1)	
Female	614 (48.2)	354 (52.0)	260 (43.9)	
Age, median [IQR]	13.0 [6.0; 28.0]	21.0 [7.0; 31.0]	10.0 [5.0; 23.2]	<0.001
Age groups, n (%)				<0.001
0–3 years	54 (4.2)	23 (3.4)	31 (5.2)	
3–6 years	224 (17.6)	106 (15.6)	118 (19.9)	
6–12 years	324 (25.5)	143 (21.0)	181 (30.6)	
12–18 years	81 (6.4)	38 (5.6)	43 (7.3)	
18–60 years	574 (45.1)	361 (53.0)	213 (36.0)	
≥60 years	16 (1.3)	10 (1.5)	6 (1.0)	
Number of diagnoses, mean (SD)	1.6 (0.8)	1.0 (0.0)	2.3 (0.6)	<0.001
Allergic diseases, n (%)				
AR	808 (63.5)	314 (46.1)	494 (83.4)	<0.001
AS/CVA	240 (18.9)	51 (7.5)	189 (31.9)	<0.001
AD/eczema	469 (36.8)	223 (32.7)	246 (41.6)	0.001
FA	224 (17.6)	62 (9.1)	162 (27.4)	<0.001
AC	224 (17.6)	3 (0.4)	221 (37.3)	<0.001
DA	54 (4.2)	28 (4.1)	26 (4.4)	0.914
Anaphylaxis	20 (1.6)	0 (0.0)	20 (3.4)	<0.001
Allergen sIgE positivity, n (%) ^a^	838 (85.1)	365 (79.0)	473 (90.4)	<0.001
Inhaled allergens positivity, n (%) ^b^	794 (82.2)	346 (76.2)	448 (87.5)	<0.001
Ingested allergens positivity, n (%) ^b^	181 (38.3)	56 (27.2)	125 (46.8)	<0.001
tIgE level, median [IQR]	216.0 [86.4; 519.0]	136.0 [65.8; 338.0]	359.0 [166.0; 810.0]	<0.001

Abbreviations: sIgE, specific immunoglobulin E; tIgE, total immunoglobulin E; SD, standard deviation; AR, allergic rhinitis; AS, asthma; CVA, cough variant asthma; AD, atopic dermatitis; FA, food allergy; AC, allergic conjunctive; DA, drug allergy. ^a^ During the investigation, 985 patients with allergic diseases were detected for allergen sIgE; among them, 462 only had one allergic disease and 523 were allergic multimorbidity. ^b^ During the investigation, 966 patients of all were detected for inhaled allergen sIgE; among them, 454 had one allergic disease and 512 were allergic multimorbidity. Overall, 473 patients were detected for ingested allergen sIgE, with 206 having a single entity and 267 having allergic multimorbidity.

**Table 2:** Comparison of sex difference between different allergic conditions.

Allergic Conditions	All Patients (n)	Male n (%)	Female n (%)	*p* Value
Any single allergy disease	681	327 (48.0)	354 (52.0)	0.005
Allergic multimorbidities	592	332 (56.1)	260 (43.9)	
AR	314	180 (57.3)	134 (42.7)	0.841
AR multimorbidities	494	288 (58.3)	206 (41.7)	
AS/CVA	51	19 (37.3)	32 (62.7)	0.001
AS/CVA multimorbidities	189	120 (63.5)	69 (36.5)	
AD/eczema	223	93 (41.7)	130 (58.3)	0.004
AD/eczema multimorbidities	246	136 (55.3)	110 (44.7)	
FA	62	25 (40.3)	37 (59.7)	0.071
FA multimorbidities	162	89 (54.9)	73 (45.1)	
AC	3	1 (33.3)	2 (66.7)	0.610
AC multimorbidities	221	115 (52.0)	106 (48.0)	
DA	28	9 (32.1)	19 (67.9)	0.661
DA multimorbidities	26	6 (23.1)	20 (76.9)	
anaphylaxis	0	0 (0.0)	0 (0.0)	-
Anaphylaxis multimorbidities	20	11 (55.0)	9 (45.0)	

Abbreviations: AR, allergic rhinitis; AS, asthma; CVA, cough variant asthma; AD, atopic dermatitis; FA, food allergy; AC, allergic conjunctive; DA, drug allergy.

**Table 3:** Comparison of inhaled allergen sensitization between allergic multimorbidities against a single entity in four allergic diseases.

Allergens	AR, n (%)		AS/CVA, n (%)		AD/Eczema, n (%)		FA, n (%)	
	Entity	Multimorbidity	Entity	Multimorbidity	Entity	Multimorbidity	Entity	Multimorbidity
Cockroaches								
Negative	140 (83.3)	242 (81.2)	21 (100.0)	79 (79.8)	69 (92.0)	106 (70.7)	14 (48.3)	53 (53.0)
Positive	28 (16.7)	56 (18.8)	0 (0.0)	20 (20.2)	6 (8.0)	44 (29.3)	15 (51.7)	47 (47.0)
Dander								
Negative	175 (85.0)	246 (84.2)	17 (77.3)	94 (84.7)	16 (94.1)	56 (77.8)	10 (100.0)	53 (88.3)
Positive	31 (15.0)	46 (15.8)	5 (22.7)	17 (15.3)	1 (5.9)	16 (22.2)	0 (0.0)	7 (11.7)
*D.pteronyssinus*								
Negative	52 (19.5)	57 (13.1)	18 (52.9)	14 (8.5)	51 (51.0)	34 (17.0)	13 (36.1)	33 (24.4)
Positive	215 (80.5)	379 (86.9)	16 (47.1)	150 (91.5)	49 (49.0)	166 (83.0)	23 (63.9)	102 (75.6)
*D.farinae*								
Negative	50 (18.7)	52 (12.0)	19 (55.9)	14 (8.6)	51 (50.5)	32 (16.1)	11 (30.6)	32 (23.7)
Positive	217 (81.3)	383 (88.0)	15 (44.1)	149 (91.4)	50 (49.5)	167 (83.9)	25 (69.4)	103 (76.3)
Fungi								
Negative	162 (97.0)	250 (93.6)	18 (100.0)	79 (90.8)	20 (100.0)	60 (85.7)	5 (83.3)	58 (96.7)
Positive	5 (3.0)	17 (6.4)	0 (0.0)	8 (9.2)	0 (0.0)	10 (14.3)	1 (16.7)	2 (3.33)
Pollen								
Negative	148 (94.9)	197 (95.2)	24 (100.0)	68 (91.9)	10 (90.9)	48 (96.0)	5 (100.0)	23 (85.2)
Positive	8 (5.1)	10 (4.8)	0 (0.0)	6 (8.1)	1 (9.1)	2 (4.0)	0 (0.0)	4 (14.8)

Abbreviations: AR, allergic rhinitis; AS, asthma; CVA, cough variant asthma; AD, atopic dermatitis; FA, food allergy.

**Table 4:** Comparison of ingested allergen sensitization between allergic multimorbidities against a single entity in four allergic diseases.

Allergens	AR, n (%)		AS/CVA, n (%)		AD/Eczema, n (%)		FA, n (%)	
	Entity	Multimorbidity	Entity	Multimorbidity	Entity	Multimorbidity	Entity	Multimorbidity
Egg white								
Negative	48 (96.0)	135 (84.9)	10 (100.0)	46 (85.2)	68 (97.1)	98 (74.2)	26 (74.3)	60 (57.7)
Positive	2 (4.0)	24 (15.1)	0 (0.0)	8 (14.8)	2 (2.9)	34 (25.8)	9 (25.7)	44 (42.3)
Peanut								
Negative	5 (83.3)	30 (96.8)	1 (100.0)	8 (88.9)	6 (100.0)	21 (87.5)	9 (100.0)	29 (82.9)
Positive	1 (16.7)	1 (3.2)	0 (0.0)	1 (11.1)	0 (0.0)	3 (12.5)	0 (0.0)	6 (17.1)
Soybean								
Negative	6 (100.0)	22 (95.7)	2 (100.0)	5 (100.0)	2 (100.0)	18 (85.7)	8 (100.0)	27 (90.0)
Positive	0 (0.0)	1 (4.3)	0 (0.0)	0 (0.0)	0 (0.0)	3 (14.3)	0 (0.0)	3 (10.0)
Wheat								
Negative	38 (97.4)	121 (93.1)	15 (100.0)	35 (85.4)	69 (98.6)	106 (86.9)	30 (93.8)	76 (76.0)
Positive	1 (2.6)	9 (6.9)	0 (0.0)	6 (14.6)	1 (1.4)	16 (13.1)	2 (6.2)	24 (24.0)
Shrimp								
Negative	28 (96.6)	99 (78.6)	14 (100.0)	32 (74.4)	69 (97.2)	84 (68.3)	18 (50.0)	57 (54.8)
Positive	1 (3.4)	27 (21.4)	0 (0.0)	11 (25.6)	2 (2.8)	39 (31.7)	18 (50.0)	47 (45.2)
Milk								
Negative	45 (83.3)	127 (78.4)	9 (75.0)	45 (78.9)	63 (87.5)	94 (70.7)	27 (77.1)	65 (61.9)
Positive	9 (16.7)	35 (21.6)	3 (25.0)	12 (21.1)	9 (12.5)	39 (29.3)	8 (22.9)	40 (38.1)
Crab								
Negative	28 (100.0)	104 (82.5)	14 (100.0)	34 (77.3)	70 (100.0)	91 (74.6)	19 (54.3)	63 (60.0)
Positive	0 (0.0)	22 (17.5)	0 (0.0)	10 (22.7)	0 (0.0)	31 (25.4)	16 (45.7)	42 (40.0)

Abbreviations: AR, allergic rhinitis; AS, asthma; CVA, cough variant asthma; AD, atopic dermatitis; FA, food allergy.

## Data Availability

All data generated or analyzed during this study are included in this article and its supplementary material files. Further inquiries can be directed to the corresponding author.

## Supplementary Materials

The following supporting information can be downloaded at: https://www.mdpi.com/article/10.3390/jcm12062226/s1, Figure S1: Phenotype of allergic conditions in the study; Figure S2: Distribution pattern of allergic diseases by month; Table S1: Diagnostic criteria of allergic diseases; Table S2: Drug allergy in 54 patients; Table S3: Comparison of allergen sensitization between allergic multimorbidities against a single entity in AC, DA and anaphylaxis.
