# Supplementary material for: Multimorbidity of Allergic Conditions in Urban Citizens of Southern China: A Real-World Cross-Sectional Study
Source: J Clin Med. 2023 Mar 13;12(6):2226. doi: 10.3390/jcm12062226 (PMC10059830; doi:10.3390/jcm12062226)
Supplement: Supplementary file 1 [file jcm-12-02226-s001.zip › jcm-2186867-supplementary.pdf]

Supplementary materials

Table S1 Diagnostic criteria of allergic diseases

Allergic conditions	Diagnostic criteria
allergic rhinitis	The inclusion criteria were as follows:<br>1. Symptoms: two or more symptoms, such as runny nose, nasal itching or nasal congestion, persist or accumulate for more than 1 hour every day, and may be accompanied by eye symptoms such as tearing, eye itching and eye redness;<br>2. Signs:nasal mucosa is pale, and/or edematous, and/or nasal cavity is watery;<br>3. Allergen detection: at least one allergen SPT and/or serum specific lgE are positive, or nasal provocation test is positive.
asthma	A. Adult：<br>1. Clinical symptoms and signs of typical asthma: (1) recurrent wheezing, shortness of breath, with or without chest tightness or cough, frequent at night and in the morning, often associated with exposure to allergens, cold air, physical and chemical stimulation, upper respiratory tract infection, exercise, etc; (2) During the attack and some uncontrolled chronic persistent asthma, scattered or diffuse wheezing sounds can be heard in both lungs, and the expiratory phase is prolonged; (3) The above symptoms and signs can be relieved by treatment or by themselves.<br>2. Objective examination of variable airflow restriction: (1) positive bronchodilation test (after inhalation of bronchodilator, FEV1 increased by>12%, and the absolute value of FEV1 increased by>200 ml); Or after 4 weeks of anti-inflammatory treatment, FEV1 increased by more than 12% compared with the baseline value, and the absolute value of FEV1 increased by more than 200 ml (excluding respiratory tract infection). (2) Bronchial provocation test was positive; Generally, the inhalation stimulant is methacholine or histamine. Generally, FEV1 decreases by ≥ 20% after inhalation of the stimulant, and the judgment result is positive, indicating the existence of airway hyperreactivity. (3) The average daily diurnal variation rate of peak expiratory flow (PEF) e(the sum of diurnal variation rates of PEF for at least 7 consecutive days/total days 7)>10%, or the weekly variation rate of PEF {(highest PEF value in 2 weeks - lowest PEF value)/ [(highest PEF value in 2 weeks+lowest PEF) x1/2] x100%}>20%.<br>It can be diagnosed as asthma if it meets the above symptoms and signs, has any of the objective examinations of airflow restriction, and excludes wheezing, shortness of breath, chest tightness and cough caused by other diseases.<br>B. Children：<br>The diagnostic criteria of asthma in children is mainly based on respiratory symptoms, signs and pulmonary function tests, which confirm the existence of variable expiratory airflow restriction, and exclude other diseases that can cause related symptoms.<br>1. Repeated wheezing, coughing, shortness of breath, or chest tightness are mostly related to exposure to allergens, cold air, physical and chemical stimuli, respiratory tract infections, exercise, and hyperventilation, which often occur or worsen at night and/or in the early morning.<br>2. During the attack, scattered or diffuse wheezing sounds to auscultation bilaterally, mainly in the expiratory phase, and the expiratory phase is prolonged.

	3. The above symptoms and signs are effectively or spontaneously relieved by anti asthma treatment.<br>4. Except wheezing, coughing, shortness of breath and chest tightness caused by other diseases.<br>5. Those with atypical clinical manifestations (such as no obvious wheezing) should have at least one of the following criteria：<br>(1) Confirmed with reversible airflow restriction:<br>① Positive bronchodilation test: The forced expiratory volume in the first second (FEV1) increased by≥ 12% 15 min after inhalation β2 receptor agonist;<br>② Improvement of pulmonary ventilation function after anti-inflammatory treatment : FEV1 increased by ≥ 12% after 4-8 weeks of inhaled glucocorticoid and/or antileukotriene treatment;<br>(2) Bronchial provocation test was positive;<br>(3) Maximum peak expiratory flow (PEF) diurnal variability (continuous monitoring for 2 weeks) ≥ 13%.<br>Those meeting the criteria 1 to 4 or 4 and 5 can be diagnosed as asthma.<br>C. Diagnostic clues of asthma in children under 6 years old: At present, there is no specific detection that can be used as the diagnostic criteria for asthma in children under 6 years old. Therefore in this study, for patients with atypical clinical manifestations, clinicians mainly evaluate the possibility of children developing persistent asthma according to the frequency and severity of symptoms/attacks and whether there are risk factors for asthma, so as to determine whether long-term controlled treatment is needed, and further support or exclude the diagnosis of asthma according to the treatment response.In clinical practice, we also evaluate the risk of persistent asthma in young children with asthma by using the modified asthma predictive index and asthma prediction tool. Asthmatic children are highly suggestive of the diagnosis of asthma if they have the following clinical characteristics: (1) More frequent paroxysmal wheezing once a month; (2) Active cough or wheezing; (3) Intermittent nocturnal cough caused by non-viral infection; (4) The symptoms of wheezing continued after 3 years of age; (5) Anti-asthmatic treatment is effective, but it relapses after drug withdrawal.
cough variant asthma	Adult: Cough as the only or main symptom but no typical asthma symptoms and signs such as wheezing and shortness of breath, and has any of the objective tests of variable airflow restriction, except cough caused by other diseases， and which is effective by treated as asthma.<br>Children:<br>1. Cough lasts for more than 4 weeks, often occurs or worsens during exercise, at night and/or in the early morning, mainly with dry cough, without wheezing;<br>2. There is no sign of infection, or it is invalid after a long period of antibiotic treatment;<br>3. Diagnostic treatment of anti asthma drugs is effective;<br>4. Eliminate chronic cough caused by other reasons;<br>5. The bronchial provocation test positive and/or PEF daily variation rate (continuous monitoring for 2 weeks) ≥ 13%;<br>6. Allergic disease history or positive allergen test of individuals or first and second degree relatives.

	Items 1-4 are the necessary criteria for diagnosis.
atopic dermatitis /eczema	Atopic dermatitis was defined according to Williams criteria in this study：<br>1.Main criteria: skin pruritus;<br>2.Secondary criteria: (1) history of flexor involvement, including cubital fossa, popliteal fossa, anterior ankle, and neck (children under 10 years old, including cheek rash); (2)History of asthma or allergic rhinitis(or there is a history of atopic disease in the first-degree relatives of children under 4 years old); (3) History of systemic skin dryness in recent years; (4) Eczema on the flexed side (eczema on the cheek/forehead and extremities of children under 4 years old); (5) Come on before the age of 2 years (applicable to patients>4 years old).<br>Diagnostic criteria: main criteria+3 or more secondary criteria
food allergy	An adverse reaction to food mediated by an immunologic mechanism, involving specific IgE (IgE-mediated), cell-mediated mechanisms (non-IgE-mediated) or both IgE- and cell-mediated mechanisms (mixed IgE- and non-IgE-mediated).The clinical presentation of food allergy involves a large spectrum of symptoms ranging from skin (urticaria, angioedema, atopic ezema/dermatitis), gastrointestinal (i.e., vomiting, colic, abdominal pain, diarrhea, constipation), respiratory (rhinorrhea, sneezing, cough, dyspnea) to circulatory (cardiovascular collapse). In this study, clinicians make diagnosis through a careful dietary history detailed medical history either alone or in combination with skin prick tests (SPT) or serum-specifific IgE (sIgE) blood tests, or oral food challenge (OFC). Evidence of IgE sensitization to common food and appropriate aeroallergens can support a diagnosis of food allergy in conjunction with clinical history and/or food challenge. In the presence of a suggestive history, a negative SPT or sIgE needs to be interpreted with caution particularly as these are expected in non-IgE-mediated food allergy.If clinical history with SPT and/or sIgE results is not highly predictive, an OFC is required.
allergic conjunctivitis	The clinical diagnostic criteria of allergic conjunctivitis should meet the following two necessary conditions at the same time.<br>1. Symptoms: Eye itching may be accompanied by foreign body sensation, and conjunctival sac secretion increase.<br>2. Physical signs: At least one characteristic of conjunctival congestion, or conjunctival papilla or corneal specific lesions was found.<br>In the laboratory examination, eosinophils were found in conjunctival scraping, which is more helpful for the diagnosis of allergic conjunctivitis.
drug allergy	Drug allergy refers to the hypersensitivity reaction caused by drugs with clear characteristics of immunological mechanism. According to the relationship between clinical symptoms and potential immune mechanism, drug allergy is divided into four categories according to Coombs and Gell classification:<br>1. Type I hypersensitivity is mediated by IgE. This type of hypersensitivity is usually typical of quick-type hypersensitivity, manifested by urticaria, severe allergic reaction and asthma.<br>2. Type II hypersensitivity based on immunoglobulin IgG (which may also be involved by IgM, but mainly by IgG) or complement mediated cytotoxicity, the main clinical symptom is abnormal peripheral blood cells.

	3. The damage of immune complex mediated by IgG/IgM, complement or FcR leads to type III hypersensitivity reaction, which is mainly manifested as vasculitis.<br>4. Type IV hypersensitivity is mainly mediated by T cells, which are often classified as delayed type hypersensitivity.<br>Drug allergy is mainly skin manifestations, but other organs (such as heart, liver, etc.) or multi-system injuries are also common in clinical practice. Because the classification of the mechanism of drug hypersensitivity is relatively complex, currently it is mainly divided into immediate and delayed type hypersensitivity according to the time of clinical reaction. Instantaneous hypersensitivity usually refers to that the time interval from medication to symptom onset is within 1~6 hours, and some patients can occur immediately and reach the peak within half an hour. Delayed hypersensitivity reaction is that the onset time of symptoms after medication is usually more than 6 hours to several days, or even within months. In this study, the specialist made a comprehensive judgment based on the clinical manifestation of drug allergy, the patient's basic disease and concomitant factors, the characteristics of sensitized drugs, the patient's basic information, past drug allergy history and family allergy history.
anaphylaxis	Anaphylaxis should be highly suspected if one of the following two clinical criteria is met：<br>1. Symptoms of skin and/or mucous membrane tissue (such as generalized urticaria, pruritus or redness, lip - tongue - uvula swelling) with acute attack (minutes to hours) and at least one of the following criteria:<br>(1) Respiratory system symptoms (such as dyspnea, wheez-bronchospasm, wheezing, decreased peak expiratory flow rate, hypoxemia).<br>(2) Decreased blood pressure or related terminal organ dysfunction symptoms [such as hypotonia (fainting), syncope, urinary incontinence].<br>(3) Severe gastrointestinal symptoms (such as severe abdominal spastic pain, repeated vomiting), especially in the case of exposure.<br>After food allergen<br>2. Acute attack of hypotension or bronchospasm or laryngeal symptoms (minutes to hours) after exposure to the patient's known or highly probable allergens, even without typical skin symptoms.

Table S2 Drug allergy in 54 patients

Drugs	number of patients (%)
Antibiotics	46 (85.2)
non-steroidal anti-inflammatory drugs	5 (9.3)
contrast agents	4 (7.4)
antiepileptic drugs	2 (3.7)
others	5 (9.3)

Table S3 Comparison of allergen sensitization between allergic multimorbidities against a single entity in AC, DA and anaphylaxis

Allergens	AC		DA		anaphylaxis	
	Entity	Multimorbidity	Entity	Multimorbidity	Entity	Multimorbidity
**Inhaled allergens, n (%)**						
Cockroaches						
Negative	0 (0.0)	122 (80.8)	4 (100.0)	8 (57.1)	0 (0.0)	3 (27.3)
Positive	0 (0.0)	29 (19.2)	0 (0.0)	6 (42.9)	0 (0.0)	8 (72.7)
Dander						
Negative	0 (0.0)	126 (84.0)	1 (100.0)	8 (100.0)	0 (0.0)	4 (100.0)
Positive	0 (0.0)	24 (16.0)	0 (0.0)	0 (0.0)	0 (0.0)	0 (0.0)
D.pteronyssinus						
Negative	0 (0.0)	25 (12.5)	5 (100.0)	5 (27.8)	0 (0.0)	5 (41.7)
Positive	0 (0.0)	175 (87.5)	0 (0.0)	13 (72.2)	0 (0.0)	7 (58.3)
D.farinae						
Negative	0 (0.0)	21 (10.5)	5 (100.0)	5 (27.8)	0 (0.0)	4 (33.3)
Positive	0 (0.0)	179 (89.5)	0 (0.0)	13 (72.2)	0 (0.0)	8 (66.7)
Fungi						
Negative	0 (0.0)	144 (93.5)	0 (0.0)	8 (100)	0 (0.0)	3 (100.0)
Positive	0 (0.0)	10 (6.5)	0 (0.0)	0 (0.0)	0 (0.0)	0 (0.0)
Pollen						
Negative	0 (0.0)	112 (95.7)	1 (100.0)	7 (100.0)	0 (0.0)	2 (100.0)
Positive	0 (0.0)	5 (4.3)	0 (0.0)	0 (0.0)	0 (0.0)	0 (0.0)
**Ingested allergens, n (%)**						
Egg white						
Negative	0 (0.0)	46 (83.6)	4 (100.0)	9 (100.0)	0 (0.0)	9 (100.0)
Positive	0 (0.0)	9 (16.4)	0 (0.0)	0 (0.0)	0 (0.0)	0 (0.0)
Peanut						
Negative	0 (0.0)	10 (100.0)	1 (100.0)	2 (100.0)	0 (0.0)	2 (50.0)
Positive	0 (0.0)	0 (0.0)	0 (0.0)	0 (0.0)	0 (0.0)	2 (50.0)
Soybean						
Negative	0 (0.0)	6 (100.0)	1 (100.0)	1 (100.0)	0 (0.0)	4 (100.0)
Positive	0 (0.0)	0 (0.0)	0 (0.0)	0 (0.0)	0 (0.0)	0 (0.0)
Wheat						
Negative	0 (0.0)	49 (96.1)	4 (100.0)	8 (100.0)	0 (0.0)	8 (57.1)
Positive	0 (0.0)	2 (3.9)	0 (0.0)	0 (0.0)	0 (0.0)	6 (42.9)
Shrimp						
Negative	0 (0.0)	27 (75.0)	3 (100.0)	7 (87.5)	0 (0.0)	6 (54.5)
Positive	0 (0.0)	9 (25.0)	0 (0.0)	1 (12.5)	0 (0.0)	5 (45.5)

Milk						
Negative	0 (0.0)	45 (76.3)	4 (100.0)	9 (100.0)	0 (0.0)	10 (100.0)
Positive	0 (0.0)	14 (23.7)	0 (0.0)	0 (0.0)	0 (0.0)	0 (0.0)
Crab						
Negative	0 (0.0)	28 (80.0)	3 (100.0)	7 (87.5)	0 (0.0)	5 (45.5)
Positive	0 (0.0)	7 (20.0)	0 (0.0)	1 (12.5)	0 (0.0)	6 (54.5)

Abbreviations: AC, allergic conjunctive; DA, drug allergy.

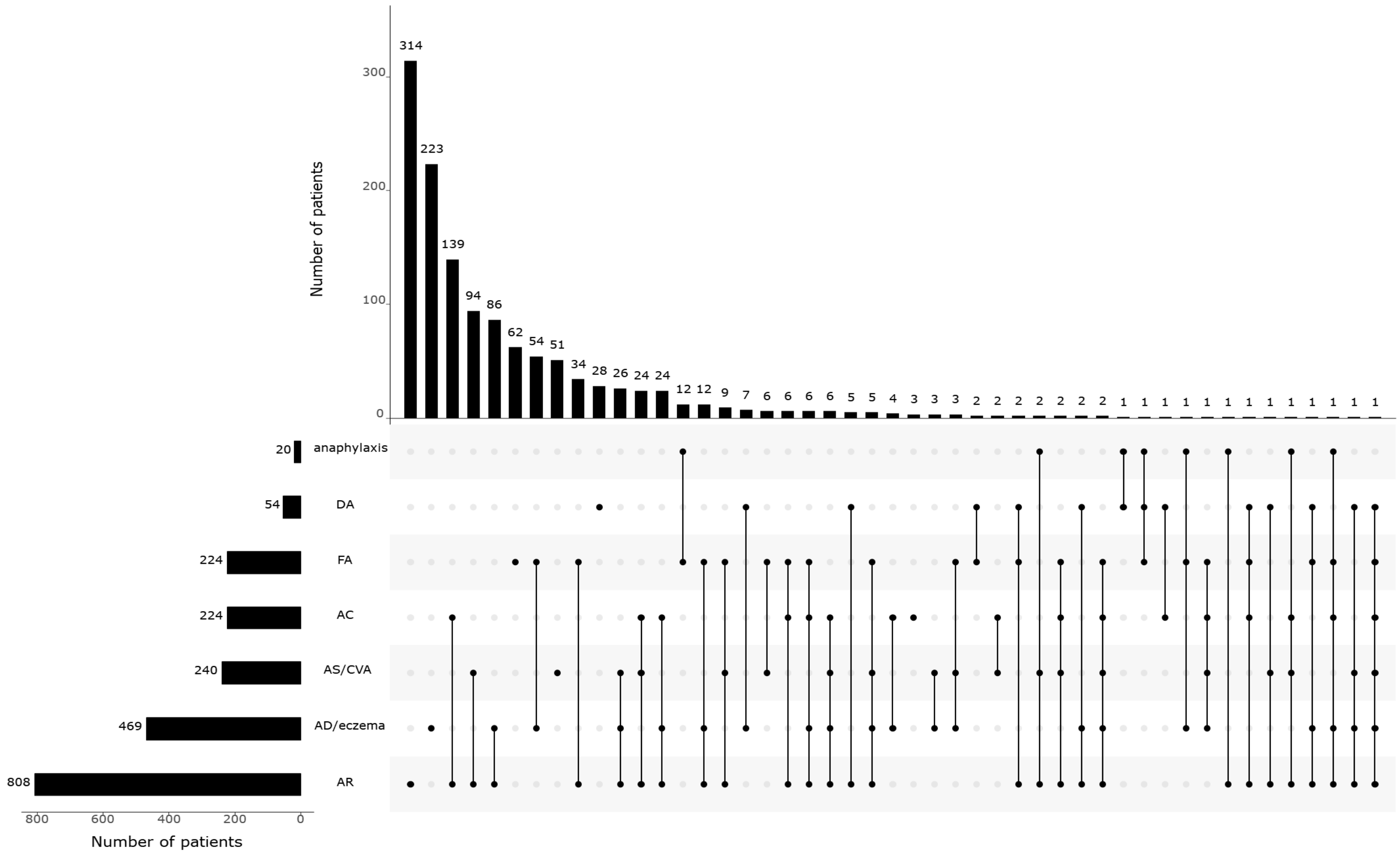

Figure S1 Phenotype of allergic conditions in the study. A total of 47 sets of allergic conditions were observed in this study.The top three allergic multimorbidities in descending order of prevalence were AR+AC (n=139), AR+AS (n=94), and AR+AD (n=86), totally accounting for 53.9 % (319 of 592) of all the allergic multimobidities. Abbreviations:AR, allergic rhinitis;AS,asthma; CVA,cough variant asthma; AD, atopic dermatitis; FA, food allergy; AC, allergic conjunctive; DA, drug allergy.

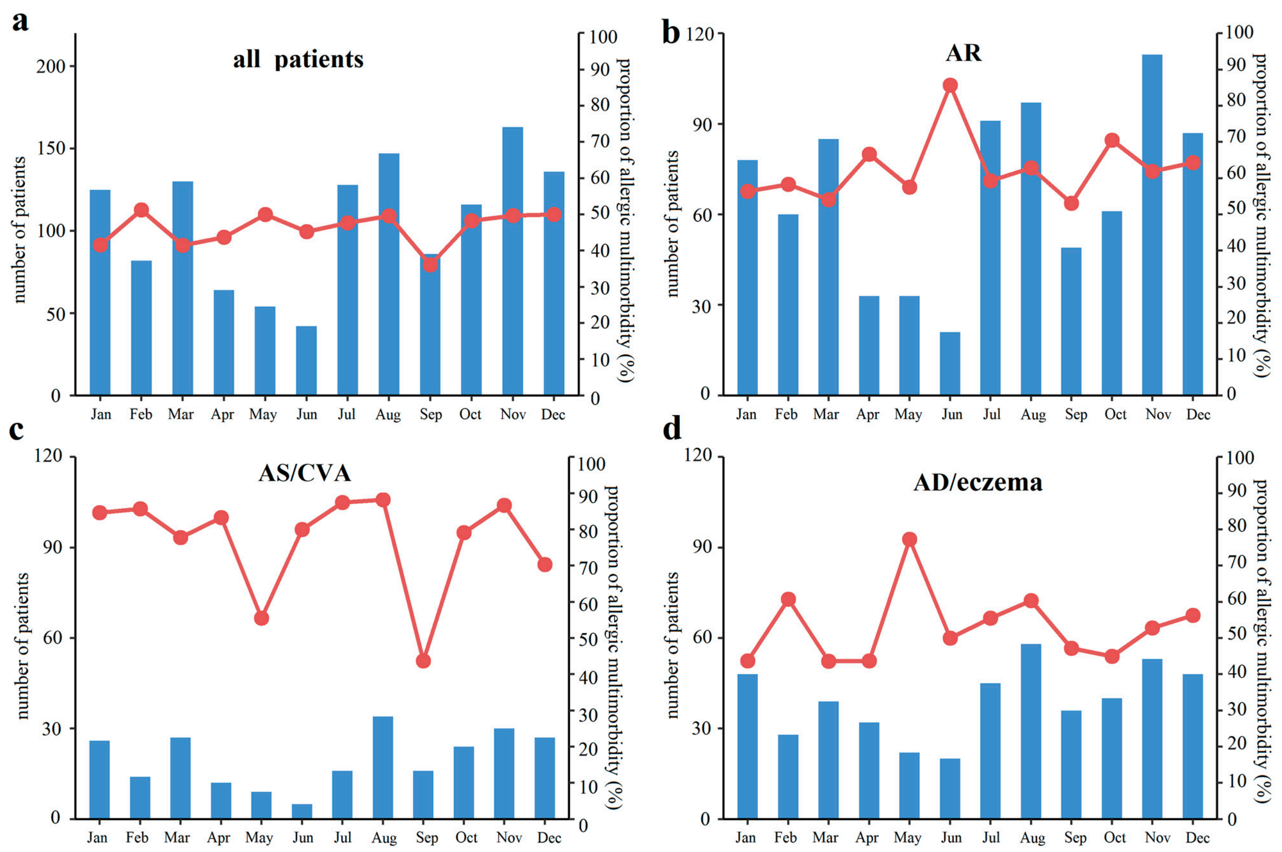

Figure S2 Distribution pattern of allergic diseases by month. The number of people diagnosed with allergic diseases each month was stable, but slightly lower from April to June. No significant differences in the frequency of allergic multimorbidities (a) were observed throughout the year, as well as between any airway (b,c) or skin (d) allergic multimorbidities (all p>0.05). Abbreviations: AR, allergic rhinitis; AS,asthma; CVA,cough variant asthma; AD, atopic dermatitis.
